# Personal Formularies of Primary Care Physicians Across 4 Health Care Systems

**DOI:** 10.1001/jamanetworkopen.2021.17038

**Published:** 2021-07-15

**Authors:** William Galanter, Tewodros Eguale, Walid Gellad, Bruce Lambert, Maria Mirica, John Cashy, Alejandra Salazar, Lynn A. Volk, Suzanne Falck, John Shilka, Elizabeth Van Dril, Jennie Jarrett, John Zulueta, Julie Fiskio, John Orav, Diana Norwich, Samuel Bennett, Diane Seger, Adam Wright, Jeffrey A. Linder, Gordon Schiff

**Affiliations:** 1 Department of Medicine, University of Illinois at Chicago, Chicago; 2 Department of Pharmacy Systems, Outcomes and Policy, University of Illinois at Chicago, Chicago; 3 Massachusetts College of Pharmacy, Boston; 4 Veterans Affairs Pittsburgh Healthcare System, Pittsburgh, Pennsylvania; 5 Northwestern Medicine, Chicago, Illinois; 6 Mass General Brigham, Boston, Massachusetts; 7 Department of Pharmacy Practice, University of Illinois at Chicago, Chicago; 8 Department of Psychiatry, University of Illinois at Chicago, Chicago; 9 Tufts Medical School, Boston, Massachusetts; 10 Emory School of Medicine, Atlanta, Georgia; 11 Vanderbilt University, Nashville, Tennessee; 12 Northwestern University Feinberg School of Medicine, Chicago, Illinois

## Abstract

**Importance:**

More conservative prescribing has the potential to reduce adverse drug events and patient harm and cost; however, no method exists defining the extent to which individual clinicians prescribe conservatively. One potential domain is prescribing a more limited number of drugs. Personal formularies—defined as the number and mix of unique, newly initiated drugs prescribed by a physician—may enable comparisons among clinicians, practices, and institutions.

**Objectives:**

To develop a method of defining primary care physicians’ personal formularies and examine how they differ among primary care physicians at 4 institutions; evaluate associations between personal formularies and patient, physician, and practice site characteristics; and empirically derive and examine the variability of the top 200 core drugs prescribed at the 4 sites.

**Design, Setting, and Participants:**

This retrospective cohort study was conducted at 4 US health care systems among 4655 internal and family medicine physicians and 4 930 707 patients who had at least 1 visit to these physicians between January 1, 2017, and December 31, 2018.

**Exposures:**

Personal formulary size was defined as the number of unique, newly initiated drugs.

**Main Outcomes and Measures:**

Personal formulary size and drugs used, physician and patient characteristics, core drugs, and analysis of selected drug classes.

**Results:**

The study population included 4655 primary care physicians (2274 women [48.9%]; mean [SD] age, 48.5 [4.4] years) and 4 930 707 patients (16.5% women; mean [SD] age, 51.9 [8.3] years). There were 41 378 903 outpatient prescriptions written, of which 9 496 766 (23.0%) were new starts. Institution median personal formulary size ranged from 150 (interquartile range, 82.0-212.0) to 296 (interquartile range, 230.0-347.0) drugs. In multivariable modeling, personal formulary size was significantly associated with panel size (total number of unique patients with face-to-face encounters during the study period; 1.2 medications per 100 patients), physician’s total number of encounters (5.7 drugs per 10% increase), and physician’s sex (−6.2 drugs per 100 patients for female physicians). There were 1527 unique, newly prescribed drugs across the 4 sites. Fewer than half the drugs (626 [41.0%]) were used at every site. Physicians’ prescribing of drugs from a pooled core list varied from 0% to 100% of their prescriptions.

**Conclusions and Relevance:**

Personal formularies, measured at the level of individual physicians and institutions, reveal variability in size and mix of drugs. Similarly, defining a list of commonly prescribed core drugs in primary care revealed interphysician and interinstitutional differences. Personal formularies and core medication lists enable comparisons and may identify outliers and opportunities for safer and more appropriate prescribing.

## Key Points

**Question:**

Given the potential quality and safety benefits associated with a more limited and conservative list of drugs a physician prescribes, how does the personal formulary size and use of core drugs vary among primary care physicians at 4 large health care organizations?

**Findings:**

In this retrospective cohort study of 4655 primary care physicians, 9 496 766 new prescriptions were written during a 2-year period, and for the 4 institutions, the mean personal formulary size ranged from 150 to 296. Multivariable modeling showed that personal formulary size was significantly associated with panel size, number of encounters, and physician’s sex.

**Meaning:**

This study found wide variations in physicians’ personal formularies, suggesting potential opportunities for improving physician practices associated with recommendations for more conservative prescribing.

## Teaser

This cohort study examines how primary care physicians’ personal formularies differ at 4 institutions; evaluates associations between personal formularies and patient, physician, and practice site characteristics; and examines the variability of the top 200 core drugs prescribed at the 4 sites.

## Introduction

Despite decades of efforts to improve the appropriateness and safety of medication use, clinicians continue to prescribe drugs liberally, often prescribing many more than various guidelines, evidence, or experts consider optimal or essential.^1,2,3,4,5^ Studies examining drug ordering patterns reveal widespread use of less appropriate or newer drugs, often before definitive safety or effectiveness data are available, and even in the face of well-documented harm.^6,7,8,9,10^

A model of prescribing has been developed that encourages more conservative and cautious use of drugs.^11,12,13^ It suggests 24 principles in 6 broad domains to guide prescribers in ordering fewer and safer drugs. One principle advises clinicians to prescribe a more limited number and mix of more evidence-based drugs. Clinicians who prescribe fewer drugs likely develop more expertise and familiarity with each medication, as well as a better knowledge of formulary coverage and cost to the patient, and their patients may have a lower frequency of adverse reactions and inappropriate medication use.^14,15,16,17,18,19^ However, this conservative prescribing principle has not been rigorously operationalized as a standardized metric, to our knowledge. Prior studies on the number of drugs used have not operationalized and standardized definitions of personal and core formularies based on new prescription orders, nor have they compared large numbers of primary care clinicians prescribing across multiple institutions using clinical data.^20,21^

The phrase *personal formulary* (PF) is defined as the unique drugs newly prescribed by an individual clinician.^20,21^ Defining and evaluating PFs may enable researchers, payers, and other health care stakeholders to create and measure prescribing behavior in a novel and standardized way. To compare prescribing among clinicians, practices, and organizations and to test hypotheses about the role of formulary size and content and their association with patient outcomes, it is first necessary to define a PF in a rigorous and reproducible manner. Defining clinicians’ PFs also can enable the creation of a list of core drugs prescribed in primary care.

As part of a multi-institutional conservative prescribing project, we developed a method of defining the PFs of primary care physicians (PCPs) and examined how PFs differed among PCPs at 4 health care institutions. The objectives of this study were to examine associations between PF size and patient, physician, and practice characteristics and to examine PF content associated with a pooled list of core drugs and the numbers and concentrations of prescriptions in selected drug classes, both within and across institutions.

## Methods

### Study Design and Data Sources

We conducted a retrospective cohort study using outpatient prescribing data from Brigham and Women’s Hospital (BWH) in Boston, Massachusetts; Northwestern Medicine in Chicago, Illinois; University of Illinois at Chicago; and the entire Veterans Health Administration (VA) from January 1, 2017, to December 31, 2018. We used a federated data model to avoid issues surrounding sharing of identifiable data.^22^ The study followed the Strengthening the Reporting of Observational Studies in Epidemiology (STROBE) reporting guideline for observational cohort studies. The study was approved by the BWH, Northwestern Medicine, University of Illinois at Chicago, and VA institutional review boards with physician and patient consent waived owing to the retrospective time frame and low risk.

### Study Cohort

We included internal medicine and family medicine PCPs at each institution. We excluded residents, fellows, subspecialists, and PCPs with fewer than 100 encounters during the study period. We identified all patients 18 years of age or older at each institution who had at least 1 face-to-face outpatient encounter with these PCPs during the study period.

### Physician and Patient Characteristics

The characteristics of the physicians were extracted from institutional data and included age, sex, and active months in the practice during the 2-year time period. In addition, we collected data on the total number of prescriptions written, the total number of newly initiated prescriptions, the number of patients with whom the PCPs had at least 1 face-to-face outpatient encounter, and the number of face-to-face encounters during the study period. The characteristics of the patients were extracted from the data and included age and sex. The mean age of the patients for the panel was calculated for each of the PCPs’ panel. Age was calculated as of January 1, 2017.

### Definition of PF

We defined a PCP’s PF as the set of unique drugs newly initiated by that PCP during the study period. We referred to these as *new starts*. A newly initiated drug was defined as one that was not previously prescribed for that patient, by any prescriber, during a 2-year lookback period in the institution’s database from the date of the index prescription. We excluded all prescriptions written during patients’ first primary care encounters at that institution to ensure that only true new starts were included because it was not possible to determine whether those first-encounter prescriptions were initiated by the study PCPs or represented renewals of prescriptions from another facility (ie, we could not access previous “lookback” prescription data in other facilities’ databases). We calculated PF size as the total number of unique drugs newly initiated by the PCP during the study period.

We used RxNorm unique identifiers (RxCUIs) to classify and standardize unique drug lists at each of the 4 institutions.^23,24,25^ We used 3 types of RxCUI: multiple ingredient, precise ingredient, and ingredient. In rare instances, drugs with the same ingredient and multiple precise ingredient codes were counted as separate drugs for ease of measurement and to ensure consistency across the 4 institutions (eg, metoprolol tartrate and metoprolol succinate were counted as 2 separate drugs). For some items where the drugs lacked RxCUI codes, study pharmacists (J.S., E.V.D., and J.J.) manually mapped the prescriptions to assign the codes.

We consolidated drug routes across the 4 institutions into 11 standardized categories: injection, intramuscular, intravenous, nasal, ophthalmic, otic, oral, rectal, subcutaneous, topical, and vaginal. Each unique combination of route and RxCUI was used to define unique drugs. Orders for intramuscular and intravenous routes were excluded from PCPs’ PFs, given the high likelihood that these were not being prescribed for home use. We used the VA drug classification system to identify drug classes for exclusion, such as bulk products, food, dietary supplements, medical devices, and compounded drugs (eTable 1 in the Supplement).

### PF Content

**Figure 1.**
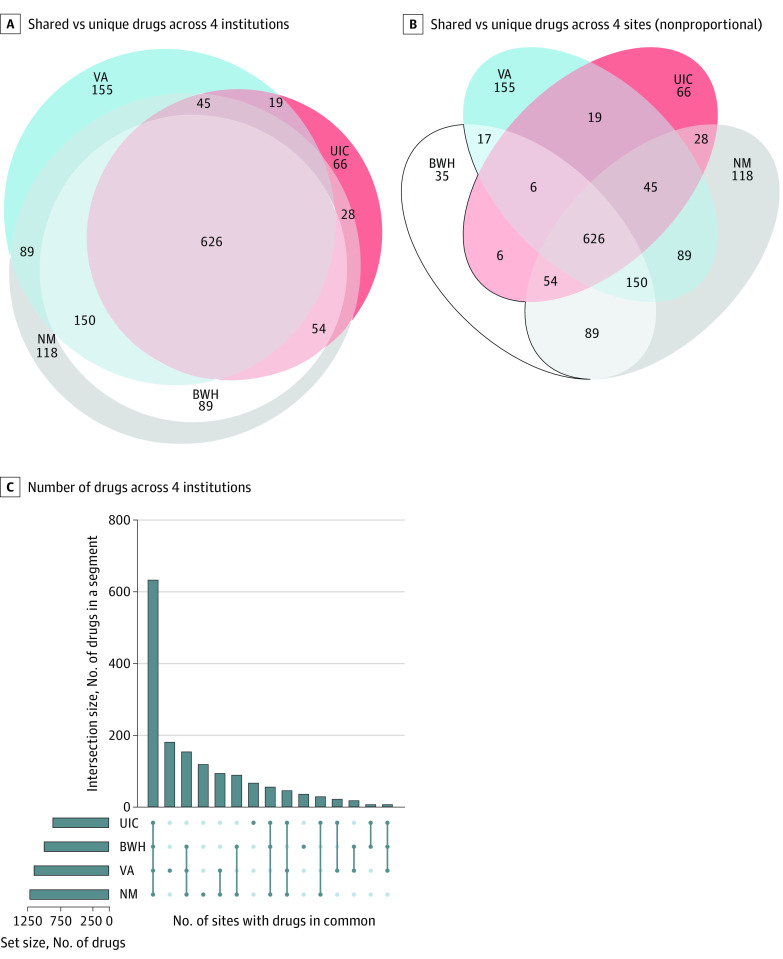
Number of Shared vs Unique Drugs at 4 Sites The Venn-Euler diagram (proportional [A] and nonproportional [B]) and the UpSetR plot (C) indicate the number of shared vs unique drugs across the 4 institutions. A, The sizes of the circles are approximately proportional to the size of the personal formulary at the institution. B, The sizes of the circles are not proportional to the sizes of the personal formulary at the institutions. C, The y-axis indicates the number of drugs in a segment; the x-axis indicates the sites with those drugs in common. The number of total medications at each site is 991 at Brigham and Women’s Hospital (BWH), 1211 at Northwestern Medicine (NM), 858 at University of Illinois at Chicago (UIC), and 1198 at the Veterans Health Administration (VA).

Venn-Euler diagrams (Figure 1A and B) were created to illustrate common overlapping and unique drugs at each site. We then empirically derived a list of core drugs prescribed by PCPs and measured how often clinicians prescribed drugs that were present on or absent from this list. Core drugs were drawn from the top 200 most frequently prescribed medications, which is consistent with other published reports.^26^ We defined the core drug lists in the 2 following ways: (1) a site-specific core list for each institution to account for institutional cultural, insurance, or formulary constraints and (2) a pooled core list from all 4 sites, with prescribing frequency weighted by the number of new prescriptions at each site. We conducted sensitivity analysis with varying cutoff values for the number of drugs in the derived core lists and used the top 200 for the analyses (the pooled core list is in eTable 2 in the Supplement). To shed additional light on the degree of conservative prescribing by individual PCPs, we measured the percentage of their prescriptions that were inside vs outside the site-specific core list and the pooled core list.

In addition to assessing the mix of each clinician’s PF in comparison with the core drug list, we calculated the total number of unique drugs clinicians ordered for commonly used drugs in that therapeutic class. From these data, we examined the concentration (vs dispersion) of total prescriptions written within that class by calculating the mean Herfindahl index^27,28,29,30^ across all clinicians for the pooled data from the 4 institutions for each of these illustrative classes of drugs.

### Statistical Analysis

We used descriptive statistics to characterize the physician and patient cohorts. We examined 2 measures of PF size: the overall number of unique drugs initiated and the number of these drugs prescribed to 2 or more patients (to minimize the association of rarely used drugs with estimates of PF size).

We used multivariable linear regression to estimate the independent association between PF size and selected covariates, including clinical site, number of encounters, number of patients, physician age and sex, percentage of female patients, and mean age of the patients in the PCP’s panel. We tested several transformations of the encounter’s variable (first-degree fractional polynomials); log transformation gave the best fit. When comparing across institutions, sites’ data on clinicians’ age and sex and patients’ mean age and sex were included in the model. Statistical tests were 2-tailed, and *P* < .05 was considered statistically significant.

The top 200 core drugs accounted for 85% to 90% of all prescriptions at the 4 institutions. We compared each PCP’s PF against these core drug lists to assess both the number of drugs in their PF that were outside this core list and the percentage of their overall prescribing that was from the core list. We performed all statistical analyses using SAS software, version 9.4 (SAS Institute Inc) and R, version 4.0.0 (R Group for Statistical Computing).

## Results

### Study Cohort

The study population included 4655 PCPs and 4 930 707 patients with at least 1 face-to-face outpatient visit with these PCPs at the 4 institutions. Overall, there were a total of 41 378 903 prescriptions written, of which 9 496 766 (23.0%) were new starts. Across the institutions, Northwestern Medicine had the highest median number of face-to-face encounters per physician (4001 [interquartile range (IQR), 2502-5377]), patients per physician (1946 [IQR, 1277-2600]), and new drug starts (2476 [IQR, 1323-3381]) (Table 1). Physicians at BWH had the lowest median number of face-to-face encounters per physician (1228 [IQR, 611-2981]), patients per physician (748 [IQR, 364-1056]), and number of new drug starts (736 [IQR, 170-1163]) compared with physicians from the other institutions, likely reflecting the fact that PCPs at this academic institution had fewer weekly sessions than community-based physicians. A total of 148 records (3.2%) did not have data on physician sex, and 150 records (3.2%) did not have data on physician age, for a total of less than 3.5% of data missing. These observations were not included in the analysis.

**Table 1.** Characteristics of Primary Care Physicians and Patients at 4 Institutions, 2017-2018

Characteristic	Median (IQR)			
	BWH	UIC	NM	VA
No. of physicians	221	63	258	4113
Personal formulary size	150 (82-212)	185 (132-228)	284 (230-347)	223 (187-268)
Physician age, y	45.9 (38.8-55.2)	44 (35.5-48.5)	51 (45.0-58.0)	53 (45.0-60.0)
Female physicians, No. (%)	124 (56.1)	37 (58.7)	139 (53.9)	1974 (48.0)
Male physicians, No. (%)	97 (43.9)	26 (41.3)	119 (46.1)	2139 (52.0)
Active months	20 (18-24)	20 (18-24)	24 (24-24)	23 (24-24)
Patients’ age in panel, mean (SD), y	53 (6)	43 (6)	49 (7)	63 (5)
No. of face-to-face encounters	1228 (611-2981)	2259 (836-3162)	4001 (2502-5377)	2757 (1706-3782)
No. of patients	748 (364-1056)	1319 (596-1865)	1946 (1277-2600)	998 (741-1280)
Total prescriptions	4785 (1381-6903)	3102 (1231-4234)	6018 (3288-8197)	9217 (4987-12 961)
New drug starts	736 (170-1163)	746 (325-1042)	2476 (1323-3381)	2067 (1097-2952)

Abbreviations: BWH, Brigham and Women’s Hospital; IQR, interquartile range; NM, Northwestern Medicine; UIC, University of Illinois at Chicago; VA, Veterans Health Administration.

### Validation by Manual Medical Record Review

To assess the accuracy of the new medication algorithm, 2 random samples of 100 medication orders each at 2 institutions were reviewed. After verification of the electronic orders and free text notes, the algorithm worked 90% of the time at BWH and 95% of the time at the University of Illinois at Chicago, for a mean (SE) accuracy score of 92.5% (2.5%).

### PF Size

Northwestern Medicine had the largest median PF size, 284 drugs (IQR, 230-347 drugs) compared with BWH, which had the lowest median PF size of 150 drugs (IQR, 82-212 drugs) (Table 1). These differences correlated with the number of encounters and patients each physician cared for. When we limited the data to drugs that were newly prescribed for at least 2 patients during the study period, the mean (SD) PF size across the sites decreased by 34% (16%). However, the full and limited PF size definitions were closely correlated, and we, therefore, elected to use the nonadjusted PF for all further analyses.

### Multivariable Analyses

**Figure 2.**
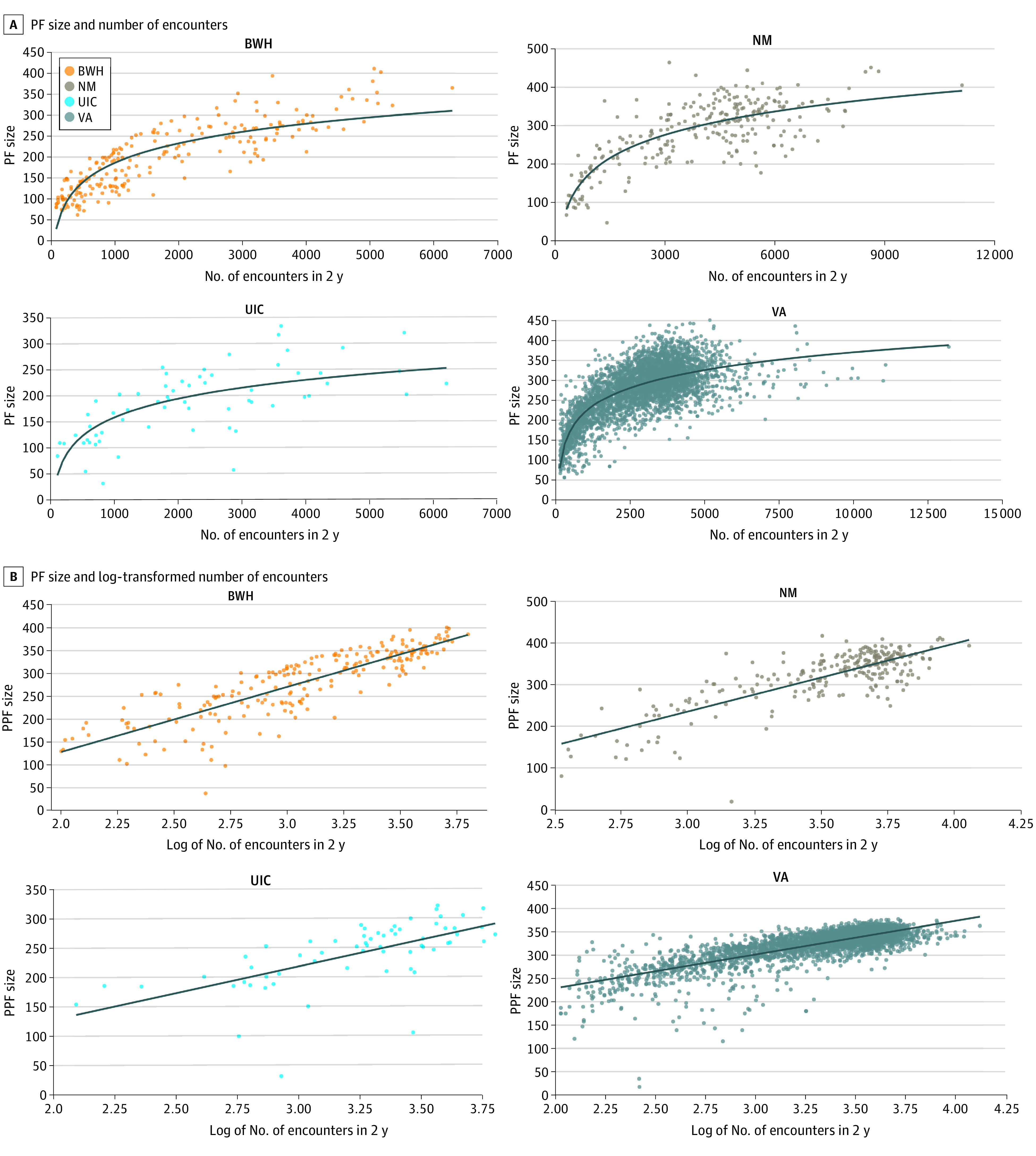
Association of Personal Formulary (PF) Size With Number of Encounters, 2017-2018 A, The y-axis indicates the size of the PF; the x-axis indicates the number of encounters. B, The y-axis indiates the size of the PF; the x-axis indicates the log-transformed number of encounters. BWH indicates Brigham and Women’s Hospital; NM, Northwestern Medicine; UIC, University of Illinois at Chicago; and VA, Veterans Health Administration.

We used linear regression to examine the role of the PCP, patient panel, and site factors in the size of the PF. The number of patient encounters was associated with PF size. A 10% increase in the number of encounters was associated with 5.7 additional drugs in a PF. For example, a physician with 3000 encounters (close to the third quartile) has, on average, 36 more drugs in their PF count than a physician with 1600 encounters (close to the first quartile). Nonetheless, there was still considerable residual variation after comparing physicians with similar numbers of encounters (Figure 2A). Physician age, mean patient age, and proportion of female patients in a physician panel were not statistically significantly associated with PF size.

Personal formulary sizes were statistically different among the 4 institutions (Table 2). After adjustement for covariates, compared with VA PCPs, BWH PCPs had approximately 46 fewer drugs in their PF, and University of Illinois at Chicago PCPs had approximately 22 fewer drugs in their PF, while Northwestern Medicine PCPs had approximately 30 more drugs in their PF. Female physicians, on average, had 6.2 fewer drugs in their PF than male physicians. Physicians actively in practice had an additional 1.5 drugs in their PF per month, and seeing 10 additional patients was associated with 1.2 more drugs in their PF.

**Table 2.** Results of Multivariable Linear Regression on the Size of Primary Care Physicians’ Personal Formularies^a^

Characteristics	Estimate (95% CI)	*P* value
Intercept	−261.09 (−284.33 to −237.86)	<.001
Center (VA is reference category)		
BWH	−37.81 (−57.35 to −18.27)	<.001
UIC	−19.47 (−31.15 to −7.79)	.001
NM	28.15 (21.38 to 34.93)	<.001
Encounters (log transformed)^b^	56.76 (53.87 to 59.65)	<.001
Active months	1.46 (0.99 to 1.92)	<.001
Physician sex (female)	−6.20 (−8.82 to −3.58)	<.001
Physician age	0.03 (−0.10 to 0.16)	.64
Patient mean age in panel	0.02 (−0.22 to 0.26)	.87
% of Female patients in panel	0.15 (−0.14 to 0.44)	.30
Physician’s mean panel size	0.012 (0.01 to 0.02)	<.001

Abbreviations: BWH, Brigham and Women’s Hospital; NM, Northwestern Medicine; UIC, University of Illinois at Chicago; VA, Veterans Health Administration.

^a^ The *R*^2^ value for the multivariable linear regression model was 0.63.

^b^ The number of encounters were log transformed after comparing several transformations (first-degree fractional polynomials); log transformation gave the best fit.

### Site and Personal Formulary Compositions

Venn-Euler diagrams were used to display drugs that were shared vs unique drugs among all possible combinations of sites. A total of 1527 unique drugs were used at least once; fewer than half (626 [41.0%]) were common to all 4 sites (Figure 1A-C).

**Figure 3.**
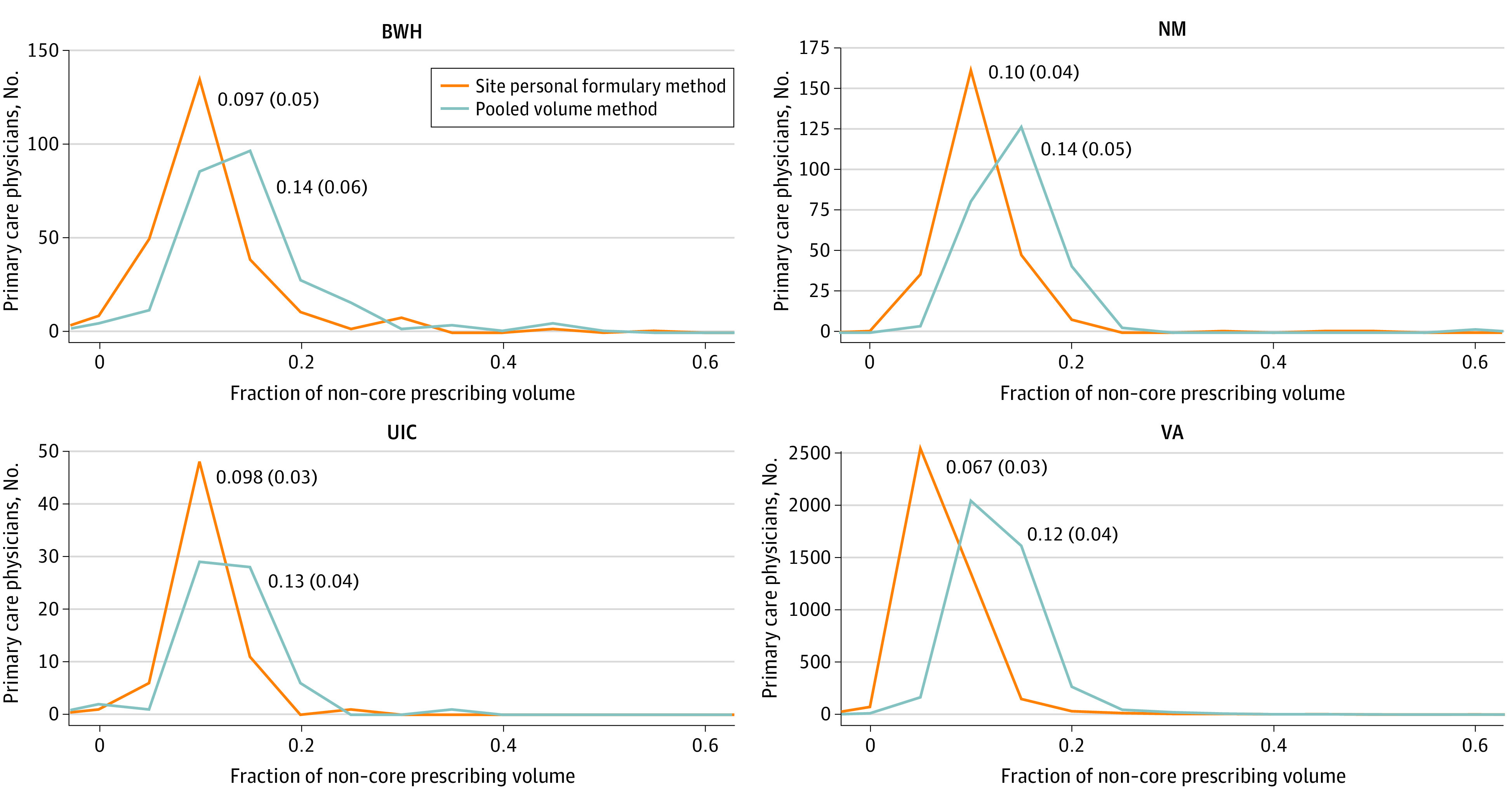
Distribution of Physicians' Prescribing Outside of Core Drug Lists, by Site BWH indicates Brigham and Women’s Hospital; NM, Northwestern Medicine; UIC, University of Illinois at Chicago; and VA, Veterans Health Administration.

The percentage of PCPs’ newly started prescriptions drawn from the core drug lists is shown in Figure 3, which illustrates that, while most clinicians write only 10% to 15% of prescriptions for drugs outside of their site core list, some prescribers are writing much higher percentages. Physicians’ prescribing of drugs from a pooled core list varied from 0% to 100% of their prescriptions.

For the 10 selected pharmaceutical classes, we found that individual clinicians varied widely in both the number of agents used within the class (eFigure 1 in the Supplement) and the distribution of agents prescribed in these classes. This demonstrates that, depending on the drug class and the prescriber, prescriptions were either more widely concentrated to a limited number of drugs or more widely dispersed, with median Herfindahl indexes ranging from 0.20 to 0.70 (eFigure 2 in the Supplement).

## Discussion

We compiled a list of unique drugs newly prescribed by PCPs at 4 institutions during a 2-year period to evaluate each physician’s PF. In addition to PF size, we also derived a core list of the top 200 drugs appearing within physicians’ PFs for each institution and overall and found marked variations among PCP physicians in both PF size and percentage of new prescriptions for core vs noncore drugs. Institutional PCPs’ median PF size ranged from 150 (IQR, 82-212) to 296 (IQR, 230-347) medications, although we found significant variations between physicians and among institutions. Applying a lens of a conservative prescribing principle that urges parsimony in the range of different drugs physicians should use in their PFs,^12^ our data suggest that some clinicians are prescribing more conservatively than others.

As expected, a key determinant of PF size is the total number of encounters and patients cared for by the physician. Logically, greater volume exposes a clinician to a wider variety of patient problems and medication indications. Performing the regression analysis, we found that the number of new prescriptions did not increase linearly but appeared to reach a saturation point that produced a better fit with the logarithm of the number of encounters, shown in Figure 2B. This saturation effect is not surprising because it may be expected that, with more encounters, the number of new situations requiring a new medication becomes lower.

However, even after controlling for encounter and patient panel size, we found significant variations in PF size. Although we were unable to control for disease case mix, we found that more than 37% of the variation in PF size remained unexplained; much of this is likely due to differing prescribing styles among the 4655 physicians and 4 institutions.

Being a female physician was also significantly associated with smaller PF size. One might assume that this might be due to a different age and sex composition in female PCPs panels. This was not the case, however, because the panel composition was controlled for and the age and sex distributions of the panels were not associated with the PF size. This is an interesting finding that may be worth further study.

Looking at the composition of each physician’s PF, we compared the mix of drugs that the clinicians prescribed. One aspect of their prescribing and PF is the extent to which they prescribed mainly from a list of core drugs used by their peers as opposed to prescribing outside of this core list. We defined the core list of drugs in a way that permitted us to compare each prescriber with their counterparts both within and outside of their institution. Again, we found significant variation in prescribing patterns, ranging from 30% to more than 90% of prescriptions being written for core drugs. Even controlling for patient volume, we can see significant differences that are unique markers of physicians’ prescribing styles. Finally, an analysis of the drugs that each clinician used in selected classes demonstrated significant variance in the choices of drugs for treating the same disease. Although we lack the ability to more rigorously adjust for the case mix of these clinicians’ patients, this exploratory look at their mix of prescriptions suggests that, to a certain extent, their larger-sized PFs may represent a less-parsimonious use of different drugs for the same condition. These findings point to opportunities for identifying outlier clinicians and providing feedback and education about ways to improve their prescribing.

Prescribing a smaller variety of drugs would permit clinicians to be more knowledgeable about dosing regimens, adverse reactions, indications, outcome data, contraindications, drug-drug interactions, and insurance coverage requirements.^31,32^ Improved medication knowledge can also facilitate the ability to engage in shared decision-making and communication regarding adverse effects and safe and effective medication use.^33^ Furthermore, this conservative prescribing philosophy is in line with a basic tenet of quality improvement theory—to reduce complexity and simplify processes.^34,35^ The idea that limiting the prescription of drugs to those that are the most essential, evidence-based, and cost-effective is the basis for the successful World Health Organization global initiative embodied in its Model List of Essential Medicines as well as for the implementation of medication formularies in various organizations and drug plans.^36,37,38,39^

### Limitations

This study has some limitations. Although, to our knowledge, this comparison of PFs across 4 institutions represents the first in its kind of analysis of clinical data that generated a large-scale comparison of clinicians and institutions, the effort faced considerable challenges. Some institutions had challenges identifying PCP prescribers at primary care clinics. Even more challenging was the standardization of drugs across the institutions, which at times required manual effort of mapping prescriptions to RxNorm RxCUIs. In addition, to exclude items such as supplies and vitamins, we also had to map each RxCUI code to a standardized classification of drugs and other products. Although we found the VA classification to be the most practical for our purposes, many items lacked RxCUI codes, and our pharmacists had to manually classify hundreds of prescriptions.

Even though using health medical record prescribing data, unlike claims prescription data, allowed us to examine finer clinical details for this and future studies, it made it more challenging to distinguish newly initiated prescriptions from renewals of chronic drugs previously prescribed at another institution. To minimize this, we excluded prescriptions written at a first PCP encounter in our 4 institutions. Review of a sample of medical records from 2 institutions showed that our algorithm for excluding previously prescribed drugs had a mean (SE) accuracy rate of 92.5% (2.5%). A repeated prescription for a short-term medication (eg, a 1-week prescription for an antibiotic) that was in fact a newly initiated prescription by that prescriber could potentially be excluded if the patient had received this medication during the 2-year lookback period. One important limitation was the lack of case-mix adjustment—something we were unable to do given the complexities, inaccuracies, and incompatibilities of problem lists and billing procedures across the 4 institutions. We did include panel mean age and proportion of female patients in the analysis and found that these panel patient attributes were not significantly associated with the PF size. We made a simplifying assumption that during a 2-year period, a PCP would encounter roughly a similar mix of patients and diseases, an assumption we believe is largely true, although clinicians may vary in the comprehensiveness of the patients they care for and the conditions they manage.^40^ Although the list of 200 core drugs that we derived represents drugs most frequently chosen for use by the PCPs, these peer-prescribing benchmarks may not necessarily mean that they are the optimal drugs of choice for the indications for which they were used. Nonetheless, these likely encompass the most essential drugs for conservative primary care prescribing. Finally, we did not measure whether a more parsimonious PF resulted in improved or worsened patient outcomes.

## Conclusions

We applied a key conservative prescribing principle, urging parsimony in a clinician’s PF size, to a large database of newly initiated prescriptions from 4 health care institutions and found significant variation between institutions and among physicians. There were significant differences in the percentage of prescriptions physicians wrote for drugs on a core list of drugs as well as variations in their prescribing in selected common primary care drug classes. Uncovering these varying prescribing patterns has the potential to help physicians and institutions compare themselves with peers and other institutions to better understand prescribing practices and identify opportunities for improving the safety, appropriateness, and cost of medication prescriptions in primary care.

## Notes

Supplement. **eTable 1.** Exclusion Classes **eTable 2.** Pooled 200 Core Drugs in Order of Use **eFigure 1.** Boxplots of the Select 10 Drug Classes **eFigure 2.** Boxplots of the Herfindahl Index for the Select 10 Drug Classes
