# Missing Funding/Support

**DOI:** 10.1001/jamanetworkopen.2021.27092

**Published:** 2021-08-19

In the Original Investigation titled “Personal Formularies of Primary Care Physicians Across 4 Health Care Systems,”^1^ published July 15, 2021, information on Funding/Support was missing, and the Role of the Funder/Sponsor paragraph needed to be revised. This article has been corrected.^1^
