# Supplementary material for: Personal Formularies of Primary Care Physicians Across 4 Health Care Systems
Source: JAMA Netw Open. 2021 Jul 15;4(7):e2117038. doi: 10.1001/jamanetworkopen.2021.17038 (PMC8283562; doi:10.1001/jamanetworkopen.2021.17038)
Supplement: Supplement. — eTable 1. Exclusion Classes eTable 2. Pooled 200 Core Drugs in Order of Use eFigure 1. Boxplots of the Select 10 Drug Classes eFigure 2. Boxplots of the Herfindahl Index for the Select 10 Drug Classes [file jamanetwopen-e2117038-s001.pdf]

Supplementary Online Content

Galanter W, Eguale T, Gellad W, et al. Personal formularies of primary care physicians across 4 health care systems. *JAMA Netw Open.* 2021;4(7):e2117038. doi:10.1001/jamanetworkopen.2021.17038

eTable 1. Exclusion Classes
eTable 2. Pooled 200 Core Drugs
eFigure 1. Boxplots of the Select 10 Drug Classes
eFigure 2. Boxplots of the Herfindahl Index for the Select 10 Drug Classes

This supplementary material has been provided by the authors to give readers additional information about their work.

© 2021 Galanter W et al. *JAMA Network Open.*

eTable 1. Exclusion Classes

1. By class in the VA classification system:
    a. Antiseptics/disinfectants (AS000)
    b. Diagnostic agents (DX000)
    c. Herbs/alternative therapies (HA000)
    d. Intrapleural (IP000)
    e. Irrigation/dialysis (IR000)
    f. Pharmaceutical aids/reagents (PH000)
    g. Miscellaneous agents (XX000)
    h. Vitamins (VT000)
2. By subclass in the VA classification system:
    a. Blood derivatives (BL500)
    b. Local anesthetics, injection (CN204)
    c. Sun protectants/sunscreens/topical (DE300)
    d. Emollients (DE350)
    e. Soaps/shampoos (DE400)
    f. Dermatologicals, Topical other (DE900)
    g. Vaccines (IM100)
    h. Toxoids (IM105)
    i. Vaccines/toxoids, other (IM109)
    j. Antivenom (IM300)
    k. Immunoglobulins (IM500)
    l. Prosthetics/supplies/devices (IN005, XA000)
    m. Anesthetics, mucosal (NT300)
    n. Lipid supplements (TN300)
    o. Amino acids/proteins (TN501, TN503)
    p. Therapeutic nutrients/minerals/electrolytes, Other (TN900)

© 2021 Galanter W et al. *JAMA Network Open.*

eTable 2. Pooled 200 Core Drugs in order of use

Medication name	Route
Albuterol	inhalation
fluticasone	inhalation
budesonide/formoterol fumarate	inhalation
insulin glargine,	subcutaneous
fluticasone propionate/salmeterol	inhalation
insulin aspartame	subcutaneous
pregabalin	oral
sitagliptin	oral
dulaglutide	subcutaneous
apixaban	oral
beclomethasone	inhalation
liraglutide	subcutaneous
insulin detemir	subcutaneous
insulin lispro	subcutaneous
emtricitabine/tenofovir (TDF)	oral
estrogens, conjugated	vaginal
mometasone	inhalation
saxagliptin	oral
atorvastatin	oral
fluticasone	nasal
azithromycin	oral
omeprazole	oral
Amlodipine	oral
lisinopril	oral
metoprolol	oral
metformin	oral
cyclobenzaprine	oral
ibuprofen	oral
gabapentin	oral
prednisone	oral
sildenafil	oral
hydrochlorothiazide	oral
amoxicillin/potassium clavulanate	oral
aspirin	oral
losartan	oral
triamcinolone	topical
benzonatate	oral
naproxen	oral
levothyroxine	oral
sulfamethoxazole/trimethoprim	oral

© 2021 Galanter W et al. *JAMA Network Open.*

codeine phosphate/guaifenesin	oral
ciprofloxacin	oral
amoxicillin	oral
tramadol	oral
ranitidine	oral
fluconazole	oral
tamsulosin	oral
simvastatin	oral
sertraline	oral
diclofenac	topical
lorazepam	oral
docusate	oral
hydrocodone/acetaminophen	oral
acetaminophen	oral
valacyclovir	oral
cetirizine	oral
loratadine	oral
methylprednisolone	oral
hydrocortisone	topical
trazodone	oral
pantoprazole	oral
lidocaine	topical
bupropion	oral
metronidazole	oral
oseltamivir	oral
ferrous sulfate	oral
furosemide	oral
alprazolam	oral
rosuvastatin	oral
cephalexin	oral
glipizide	oral
meloxicam	oral
ondansetron	oral
zolpidem	oral
ketoconazole	topical
clotrimazole	topical
potassium chloride	oral
lisinopril/hydrochlorothiazide	oral
levofloxacin	oral
pravastatin	oral
montelukast	oral
polyethylene glycol 3350	oral
allopurinol	oral

© 2021 Galanter W et al. *JAMA Network Open.*

diclofenac	oral
meclizine	oral
finasteride	oral
nicotine	topical
famotidine	oral
carvedilol	oral
tadalafil	oral
duloxetine	oral
fluoxetine	oral
amitriptyline	oral
propranolol	oral
citalopram	oral
mupirocin	topical
acetaminophen with codeine	oral
norgestimate-ethinyl estradiol	oral
atenolol	oral
clonazepam	oral
atovaquone/proguanil HCl	oral
clobetasol	topical
diazepam	oral
hydroxyzine	oral
erythromycin base	ophthalmic
spironolactone	oral
venlafaxine	oral
methocarbamol	oral
norethindrone ac-ethinyl estradiol	oral
clindamycin	topical
chlorthalidone	oral
guaifenesin	oral
clindamycin	oral
nystatin	topical
nitroglycerin	oral
baclofen	oral
clotrimazole/betamethasone dip	topical
losartan/hydrochlorothiazide	oral
nicotine	oral
guaifenesin/dextromethorphan	oral
metronidazole	vaginal
penicillin V	oral
azelastine	nasal
oxybutynin	oral
magnesium oxide	oral
ipratropium/albuterol sulfate	inhalation

© 2021 Galanter W et al. *JAMA Network Open.*

nifedipine	oral
carbamide peroxide	otic
terazosin	oral
celecoxib	oral
alendronate	oral
acyclovir	oral
dicyclomine	oral
colchicine	oral
triamterene/hydrochlorothiazide	oral
clarithromycin	oral
valsartan	oral
nitrofurantoin	oral
diltiazem	oral
diphenhydramine	oral
esomeprazole	oral
L-norgest/e.estradiol-e.estrad	oral
mirtazapine	oral
neomycin/polymyxin B/hydrocortisone	otic
lansoprazole	oral
indomethacin	oral
estradiol	vaginal
topiramate	oral
fluocinolone	topical
tretinoin	topical
pseudoephedrine	oral
Ipratropium	nasal
metronidazole	topical
scopolamine	topical
hydralazine	oral
paroxetine	oral
capsaicin	topical
econazole	topical
ciclopirox	topical
buspirone	oral
clonidine	oral
butalbitol/acetaminophen/caffeine	oral
oxycodone HCl/acetaminophen	oral
mometasone	nasal
sennosides	oral
hydrocortisone	rectal
fexofenadine	oral
isosorbide	oral
sucralfate	oral

© 2021 Galanter W et al. *JAMA Network Open.*

olopatadine	ophthalmic
enalapril	oral
cefdinir	oral
acetazolamide	oral
fenofibrate	oral
irbesartan	oral
phenazopyridine	oral
bisacodyl	oral
simethicone	oral
nortriptyline	oral
loperamide	oral
mometasone	topical
ezetimibe	oral
warfarin	oral
glimepiride	oral
norethindrone	oral
Ipratropium	inhalation
phentermine	oral
calcium carbonate	oral
nystatin	oral
ferrous gluconate	oral
sennosides/docusate sodium	oral
permethrin	topical
etonogestrel/ethinyl estradiol	vaginal
lactulose	oral
desonide	topical
lovastatin	oral
quetiapine	oral
labetalol	oral
betamethasone	topical
donepezil	oral

© 2021 Galanter W et al. *JAMA Network Open.*

eFigure 1. Boxplots of the Select 10 Drug Classes

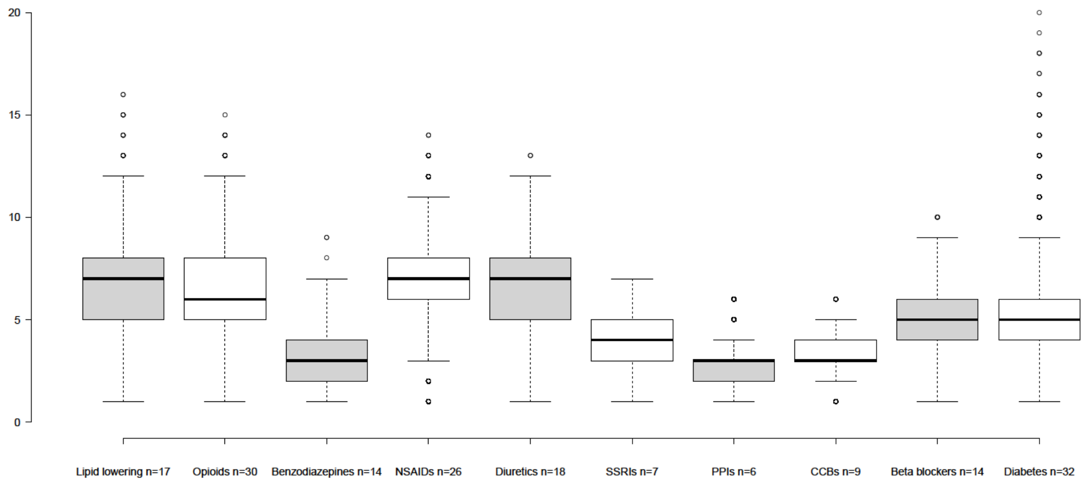

The 10 drug classes were selected for the following reasons: being the most commonly prescribed drugs in internal medicine, having multiple agents in each class, demonstrating variation in PCPs' prescribing, having old and new drugs in each class.

N next to each drug class name indicates the maximum number of drugs available in that class.

© 2021 Galanter W et al. *JAMA Network Open.*

eFigure 2. Boxplots of the Herfindahl Index for the Select 10 Drug Classes

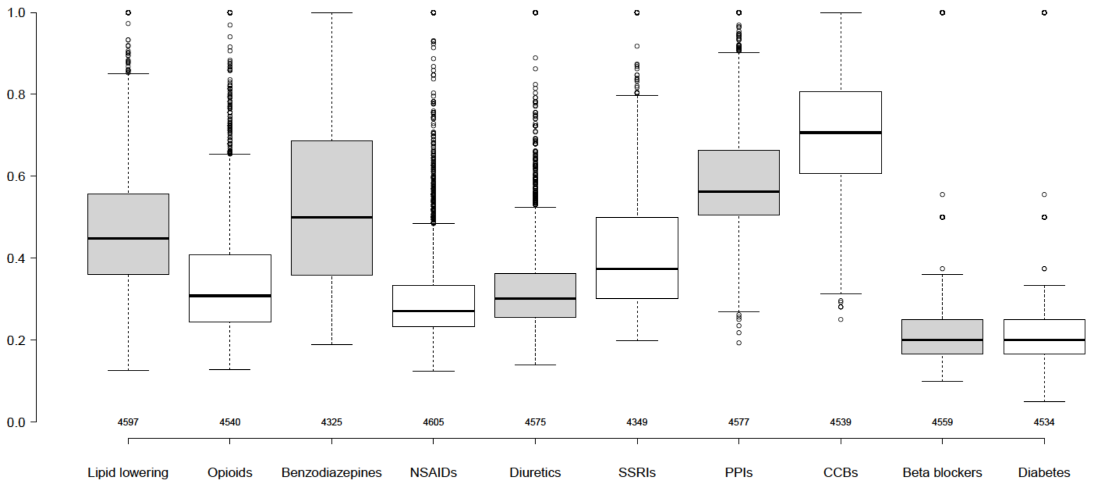

X axis shows the total number of PCPs at the 4 sites who wrote at least 1 prescription within each drug class; the Y axis shows the Herfindahl index (HHI) distribution. HHI range is 0.1 to 1.0. Although the Herfindahl indices may not be directly comparable between the different drug classes (given the varying number of drugs used in each class), each boxplot demonstrates the extent to which prescribers concentrated their prescriptions to a more limited number of drugs in the class e.g., those with a higher Herfindahl index versus more varied in their use of agents in that class, dispersing their prescriptions over a wider number of drugs resulting in a lower Herfindahl index which we observed for many of the prescribers in each of the drug illustrated classes.

© 2021 Galanter W et al. *JAMA Network Open.*
